# Beauty ideals and body positivity: a qualitative investigation of young women’s perspectives on social media content in China

**DOI:** 10.3389/fpsyg.2024.1389935

**Published:** 2024-05-20

**Authors:** Min Lang, Yiduo Ye

**Affiliations:** ^1^School of Education and Psychology, Chengdu Normal University, Chengdu, China; ^2^School of Psychology, Fujian Normal University, Fuzhou, China

**Keywords:** beauty ideal, body positivity, social media, body image, China

## Abstract

Much of the existing knowledge regarding the impact of beauty ideals and body positive social media content on women’s body image is based on the Western cultural context. This limits our understanding of the issue in other cultures, such as China, among others. Therefore, to address this gap, this study examined young Chinese women’s perspectives on beauty ideals and body positivity in social media through a qualitative investigation. Female university students in China (*N* = 24) participated in individual interviews. A thematic analysis revealed four primary themes: (1) characteristics of mainstream beauty ideals in Chinese social media; (2) impact of beauty ideals on young women; (3) perspectives on the content and roles of body positivity; (4) influences of body positive social media content on young women. These findings indicate that young Chinese women are aware of the beauty ideals in social media and their negative impact on their body image. Furthermore, young Chinese women generally expressed a favorable outlook on body positivity but noted its limitations.

## 1 Introduction

Body image concerns and related disordered eating behaviors are prevalent among young Chinese women (Zhang et al., 2018; Wu et al., 2022). Media pressure, particularly the large number of unrealistic beauty ideals, may create body image concerns in China, as well as in Western cultural settings (Jackson et al., 2020; Wu et al., 2022). Recently, the global popularity of social media has exacerbated the problem of women’s body image (Rodgers et al., 2021). Conversely, media content that challenges unattainable predominant women’s beauty ideals and encourages women to accept and appreciate their bodies regardless of their appearance, known as body positivity, has emerged in Western social media, becoming popular in Chinese social media in 2020 (Cwynar-Horta, 2016; Lang et al., 2023). Studies have identified the relationship between beauty ideals in social media and poor body image among young Chinese women, with some of them focusing on positive social media content in China; however, the perspectives of young Chinese women on such social media content remain unclear (De Valle et al., 2021; Jackson et al., 2021; Guo et al., 2022; Lang et al., 2023; Wang et al., 2023). Therefore, this study aimed to explore young Chinese women’s understanding of and perspectives on beauty ideals and body positivity in social media and reveal their cognition of the effect of body positive social media content on women’s body image.

### 1.1 Body image concerns and related behaviors among young women in China

In recent years, body image disturbances and related weight loss, dieting, and plastic surgery behaviors among young Chinese women have increased (Hong'e, 2017; Jackson et al., 2020). A study of 2,023 young Chinese women found that, although only 41 were overweight or obese, 1,484 (over 73%) reported taking action to lose weight in the past 6 months and 1,161 (over 57%) declared wanting to be thinner (Zhang et al., 2018). Similarly, a report by Guangming Online (South China Morning Post, 2020) noted that 72% of Chinese women were not satisfied with their appearance and more than 33% had undergone medical cosmetic surgery to improve their facial appearance. Another recent study showed that the incidence of eating disorders in China, particularly among adolescents (average annual percent change: males (10-14 years) = 1.22, females (10-14 years) = 1.51, males (15-19 years) = 1.11, females (15-19 years) = 1.46) and young adults (average annual percent change: males (20-24 years) = 1.06, females (20-24 years) = 1.32, males (25-29 years) = 1.15, females (25-99 years) = 1.34,), increased from 1990 to 2017 (Wu et al., 2022). Moreover, another report showed that nearly 20 million Chinese people, of whom over 87% were women and 77% were young women under 30 years of age, have undergone cosmetic surgery (New Oxygen, 2021). These findings suggest that body image is a socially significant topic in China.

### 1.2 Beauty ideals in social media and its negative influences on women’s body image

Consistent with the predictions of sociocultural theories, unattainable mainstream beauty ideals create poor body image among women in both Western and Chinese cultures (Jackson et al., 2020; De Valle et al., 2021). Western social media presents two predominant beauty ideals: thinspiration and fitspiration. Both ideals encourage women to pursue thinness and achieve it through diet and exercise (Cohen et al., 2019).

However, the beauty ideals displayed on Chinese social media include not only the pursuit of thinness but also other characteristics, such as white skin and big eyes, which are closely related to Chinese culture. In fact, in traditional Chinese literature, the most common features of idealized women’s images are smooth white skin, clear bright eyes, thick black hair, an oval face, a small cherry-like mouth, and a willow branch-like waist (Chen, 1995). Women’s beauty ideals presented in contemporary Chinese media include thinner bodies, palm-sized faces, delicate white skin, big eyes, high nose bridges, and double eyelids (Jung, 2018). A recent content analysis of beauty ideal videos on TikTok found that beauty ideals are described as natural and delicate features (big eyes, high nose bridge, oval or melon-shaped face), delicate makeup, flawless skin, and a thin body (Quan, 2021). Correspondingly, young Chinese women feel pressured not only to be thin but also generally beautiful (Jackson et al., 2020). A 12-month study found that media pressure on appearance, such as weight, facial features, and appearance preferences, explained young Chinese women’s body image concerns with fatness, facial features, and stature and interest in cosmetic surgery (Jackson et al., 2020). In addition, Asian media influenced young Chinese women’s concerns regarding appearance more than Western media (Jackson et al., 2020). Moreover, existing studies has confirmed that social media use negatively predicts young women’s body image (De Valle et al., 2021). Some correlational studies on young Chinese women have shown that social media use is significantly correlated with body image concerns (Niu et al., 2020; Sun, 2021; Yao et al., 2021).

### 1.3 Body positivity in social media and its influence on women’s body image

Body positivity has become increasingly popular in social media (Cohen et al., 2019; Lang et al., 2023). Instagram displays various women’s bodies, such as overweight women practicing yoga, plus-size women wearing fashionable clothes, and women with body image disorders sharing their struggles (Cohen et al., 2019). In China, many women show the process of removing and applying makeup and the differences between their original selfies, those taken with a beauty camera, and those modified with photo editing software. This is intended to demonstrate that the perfect selfies and ideal appearances presented in social media are not real (Lang et al., 2023).

As momentum has progressed, researchers have begun to focus on body positivity in social media. Two studies analyzed body positive posts on Instagram and found that most posts conveyed messages that aligned with the theoretical definitions of positive body images; further, several posts included appearance-focused messages (Cohen et al., 2019; Lazuka et al., 2020). A content analysis found that although over 40% of body positive posts on Chinese social media conveyed appearance-focused messages, these posts displayed substantial diversity and realistic women’s body images; most of the posts contained positive body image themes and nearly half contained self-compassion themes (Lang et al., 2023). Experimental and longitudinal studies based on Instagram indicate that viewing body positive social media content is beneficial for women’s body image, such as body satisfaction and body appreciation (Cohen et al., 2019; Tiggemann and Anderberg, 2019; Stevens and Griffiths, 2020; Fioravanti et al., 2021). Meanwhile, some studies have found that viewing body-positive social media content may also induce negative body image, such as self-objectification (Cohen et al., 2019). Overall, these findings suggest that viewing body positive social media content may be somewhat beneficial to body image (Rodgers et al., 2022). However, most of the existing studies have explored body positivity from the perspective of its impact on women’s body image. The perceptions of young women, who are the primary targets of this social media content particularly in China, regarding these posts remain unclear. Therefore, this study aimed to identify young Chinese women’s thoughts and perspectives on body positive social media content.

### 1.4 Perspectives and responses of young women to beauty ideas and body positivity

The emergence of the body positivity movement indicates that young women are aware of the negative effects of mainstream beauty ideals on their physical and mental health and are beginning to resist beauty ideals in the media (Rodgers et al., 2022). Evidence of this can be found in the existing literature. A qualitative study found that adolescents with a positive body image believe that current ideals are unrealistic and unnatural and blame the media for only showing images that are consistent with those ideals (Holmqvist and Frisén, 2012). Young Japanese women have expressed criticism and resistance to the predominant beauty ideals presented in the Japanese media, describing them as unrealistic and harmful and questioning their function in for-profit businesses (Ando et al., 2021). Furthermore, in a qualitative study of Swedish men’s and women’s journeys from negative to positive body image, participants described a crucial turning point when they found a new social context, started experiencing agency and empowerment, and began using cognitive strategies to improve their body image (Gattario and Frisén, 2019). The study also found that women felt that they had to work harder and more constantly than men to resist beauty ideals and maintain a positive body image (Gattario and Frisén, 2019).

The Body Positive movement aims to challenge mainstream beauty ideals and encourage women to accept and appreciate their bodies no matter what they are. However, whether the original intention of the campaign is fulfilled requires understanding how the movement is viewed by the target group, young women. Two recent studies examined how young people respond to body positive social media content. The results found that although body positive social media content was considered to be more helpful to women’s body image than mainstream beauty ideals, body positive content is heterogeneous, with young women having different attitudes towards different types of body positive content (Rodgers et al., 2023). To be specific, body positive content highlighting social media’s curated and unrealistic nature and posts resisting beauty ideals and embracing body appreciation was deemed helpful (Rodgers et al., 2023). Participants also stated that their perceived similarity in socio-demographic factors enhanced the potential positive effects of body image content (Rodgers et al., 2023). Conversely, posts that were less explicitly resistant to beauty ideals were viewed as less helpful (Rodgers et al., 2023). Another study found that some health professionals saw benefits in the body positivity movement, whereas others expressed concerns regarding the health of some influencers, particularly those with a larger figure (Sharp et al., 2023).

### 1.5 The present study

Given that the processes underlying the effects of social media content on women’s body image in China are poorly understood, this study aimed to examine the perspectives of young Chinese women regarding beauty ideals and body positivity. Further, it sought to gain a broader and deeper understanding of the relationship between social media content and women’s body image in China. The study targeted Chinese women aged 18–30 years, as they are the most influenced by social media images (De Valle et al., 2021). Owing to the more exploratory and preliminary nature of the topic and to gain a comprehensive understanding of the perspectives of young Chinese women, a qualitative methodology was employed. This study posed the following research questions:

1. Which characteristics do young Chinese women perceive as mainstream beauty ideals in social media?
2. What influence do young Chinese women think beauty ideals have?
3. How do young Chinese women feel about body positive social media content?
4. What influence do young Chinese women think body positivity has?

## 2 Materials and methods

### 2.1 Participants

The sample comprised young women recruited from a university in Sichuan Province, China, between December 2022 and March 2023. Participants were recruited by contacting teachers at the university and posting recruitment posters on the Internet campus wall. The posters used in both approaches were consistent, and both broadly framed the study as interview-based research focusing on social media and women’s body image. We recruited 24 female Chinese undergraduate students aged 18–21 years, with a mean age (SD) of 19.25 (0.85) years. According to Clarke and Braun (2013), 15–30 participants represent an appropriate number, which indicates that the 24 participants in our study were sufficient. The participants included 13 freshmen, 7 sophomores, and 4 juniors. All participants self-reported using social media and could describe their observations and experiences.

### 2.2 Procedure and materials

This study was approved by the Fujian Normal University Ethics Committee (No.PSY220030). After training, the first author conducted all the semi-structured interviews. The interviews were conducted in person or online according to the participants’ preferences. All participants provided written informed consent before interviews. All face-to-face interviews were conducted in a private area on the campus of the participants’ universities. All interviews lasted approximately 45 min, and some were shortened or extended according to the situation. The team developed an interview schedule to investigate the relationship between beauty ideals, body positive content on Chinese social media, and women’s body image in China. During the interviews, participants were shown body positive posts publicly available on social media as a prompt to encourage discussions on body positivity in China. Posts used for display have the following characteristics: (1) with the hashtag “#Refusing facial anxiety,” (2) publicly available, and (3) more than 100,000 likes. We chose this hashtag because body positive posts on Chinese social media are tagged with the hashtag #Refusing facial anxiety rather than #bodypositive.”This informed the knowledge and definition of the body positivity movement prior to interviews to help the participants understand and express their opinions. At the end of the interviews, participants were allowed to add additional thoughts that they considered relevant to the topic. All interviews were recorded with the participants’ consent.

Open-ended guiding questions were generated based on the research questions to obtain clearer responses. The first and second question focused on beauty ideals presented in social media, as seen and perceived by the participants, and their influences on women. The third and fourth question focused on young women’s exposure to and perception of positive social media content and its influences on women (see Supplementary material S1). Participants were compensated with RMB10 in cash for their participation in the study.

### 2.3 Data analysis

All interviews were transcribed verbatim using iFLYTEK, a confidential online subscription transcription service. As the analysis aimed to examine young Chinese women’s perspectives on beauty ideals and body positive content in social media, this study adopted a broad and critical qualitative approach, namely, thematic analysis. The analysis followed the six steps outlined by Braun and Clarke (2021) to identify meaning patterns, which is a process from a set of notes responding to data familiarization through to coding, theme development and naming. The data were analyzed using inductive and theoretical thematic analysis approaches (Forbes, 2022). The deductive approach was used to examine the data and answer specific research questions, whereas the inductive approach was used to generate themes based on data rather than being shaped by existing research agendas. Data analysis was initially deductive under the following four themes: characteristics of mainstream beauty ideals in Chinese social media; impact of beauty ideals on young women; perspectives on the content and roles of body positivity; and the influences of body positive social media content on young women. The themes in these domains were subsequently generated from the data using an inductive approach. Consequently, interviews were analyzed by the first author using NVivo Plus 12.

To manage the bias in the analysis, the second author independently coded 20% of the interviews. The researchers met to discuss the analysis and resolve any differences in the data. A complete agreement (100%) was reached between the two coders. The themes and subthemes were presented to the research team for review and discussion before reaching a consensus.

## 3 Results

All 24 participants used various types of social media. Xiao Hongshu (*n* = 21, 87.50%) and Qzone (*n* = 20, 83.33%) were the most common, followed by WeChat (*n* = 18, 75.00%), Douyin (*n* = 15, 62.50%), Weibo (*n* = 12, 50.00%), Bilibili (*n* = 9, 37.50%), and Kwai App (*n* = 2, 8.33%). Table 1 presents the themes, subthemes, and participant frequencies generated from the data. Participants could express more than one subtheme in a thematic category.

**Table 1:** Overview of themes, subthemes, and participant frequencies generated from the data.

Theme	Subtheme	Definition	Frequency; *n* (%)
Characteristics of mainstream beauty ideals on Chinese social media	Beauty ideals in traditional Chinese culture	The ideal beauty features mentioned by the participants are more frequently mentioned in traditional Chinese culture.	14 (58.33%)
	Beauty ideals in contemporary Chinese media	The ideal beauty features mentioned by participants are not mentioned in traditional Chinese culture, but are mentioned more in modern Chinese media.	10 (41.67%)
	Beauty ideals standards under the influence of Western culture	The characteristics of ideal beauty mentioned by participants are not mentioned in traditional Chinese culture, although they are mentioned in modern Chinese culture, they are more spread from Western culture. Or participants mentioned that these characteristics are more reflected in a idealized Western women.	10 (41.67%)
Impact of beauty ideals on young women	Positive impact	The positive meaning of beauty ideals mentioned by participants.	7 (29.17%)
	Negative impact	The negative effects of beauty ideals on women mentioned by participants.	21 (87.50%)
perspectives of the content and roles of body positivity	The existing problems	Existing problems with the content and role of body positivity mentioned by participants	12 (50.00%)
	The path to improvement	Participants cited ways to increase the positive impact of body positivity social media content.	11 (45.83%)
Influence of body positive social media content on young women	Positive impact	The positive effects of body positive social media content on women mentioned by participants.	20 (83.33%)
	Negative impact	The negative effects of body positive social media content on women mentioned by participants.	4 (16.67%)

### 3.1 What are the characteristics that young Chinese women perceive as mainstream beauty ideals in social media?

We found that the predominant beauty ideals in social media perceived by young Chinese women were a combination of beauty ideals in traditional Chinese culture, contemporary Chinese media, and Western culture, as well as descriptions of the overall temperament of the individual woman interviewed.

First, the standards of women’s beauty ideals in traditional Chinese culture were strongly represented in social media. The features mentioned by the participants included the overall harmony of facial features (*n* = 10, 41.67%), good face shape (*n* = 10, 41.67%), large eyes (*n* = 10, 41.67%), small mouth (*n* = 4, 16.67%), delicate and flawless skin (*n* = 14, 58.33%), white skin (*n* = 9, 37.50%), and soft and beautiful hair (*n* = 10, 41.67%). For example, Participant 7 stated:

“It is not a very pointy face. It is an oval face, the nose is high, the eyes are big, the skin is nice and tender, and the hair is fluffy.”

Participants often mentioned a small face (*n* = 5, 20.83%), double eyelids (*n* = 4, 16.67%), high nose bridge (*n* = 8, 33.33%), thin (*n* = 19, 79.17%), slender body (*n* = 12, 50.00%), slender waist (*n* = 5, 20.83%), long and well-shaped legs (*n* = 10, 41.67%), and delicate makeup (*n* = 5, 20.83%), which are women’s beauty ideals presented in contemporary Chinese media. For example, Participant 5 stated:

“She has big double eyelids, her nose is high, and her features are well-proportioned. Whenever I see those beautiful women on the Internet, I envy them, and most of them seem to be only 40–45 kg. I especially envy those who are thin because the girls are now pursuing being thinner, so I think thinner is good. I think thinner women look better.”

We calculated the body mass index of the idealized female image based on height and weight data provided by eight participants and found that five were in the underweight level and three, although within the normal range, were near the underweight level.

Moreover, beauty ideal standards influenced by Western culture, such as curves (*n* = 16, 66.67%), sexy bodies (*n* = 10, 41.67%), tight bodybuilding (*n* = 2, 8.33%), tight waist muscles (*n* = 4, 16.67%), arm muscles (*n* = 3, 12.50%), and upturned buttocks (*n* = 1, 4.17%), were mentioned frequently. “Sexy” and “curvy” were often mentioned in conjunction with “thin.” For instance, Participant 8 stated, “Although she looks a little too thin overall, she is still very sexy and curvy.”

In addition to specific body parts, other beauty standards related to the overall image of the individual, such as dressing fashionably, were mentioned by six participants. Participant 9 commented, “On the whole, they dress beautifully and fashionably.”

Furthermore, some overall characteristics of individuals were discussed. Twelve overarching impression traits were mentioned: cute, gentle and playful, loves to laugh, authentic, healthy and energetic, elegant, comfy, gentle, refreshing, intellectual, and delicate. Notably, two participants cited only “elegance,” while the remaining traits were mentioned by only one participant.”

### 3.2 What influence do young Chinese women think beauty ideals have?

The participants mentioned the negative and positive influences of the predominant beauty ideals presented in social media on young women. The positive effects were mainly reflected in (1) promoting oneself to improve one’s external image and confidence by learning makeup techniques, dressing skills, and exercise methods; (2) feeling happy upon encountering beautiful women. For instance, Participant 1 stated:

“I think the influence on me should be mostly good. I may indeed improve my dressing skills, and then I may also exercise. I just want to get a better-looking body.”

Five main categories of adverse effects were identified. First, beauty ideals cause young women to form incorrect perceptions, mainly manifested as (1) believing that their appearance is easy to change, (2) denying themselves and becoming inferior, (3) blindly following trends and losing themselves, (4) blurring the recognition of their true self, (5) thinking that body management is necessary, (6) feeling overweight despite being in the normal BMI range, (7) thinking that they must conform to the public aesthetic, and (8) thinking they have to wear makeup to go out.

“It has a big impact. After seeing social media content, I feel that my figure is not good and [I have] learned to put on makeup and dress. There are even some girls who do not go out without wearing makeup. They even think girls should wear makeup.” (Participant 3).

“If we look at it often enough, it [affects us]. After browsing this kind of social media content, we believe that we can change our facial features and body figure, too.” (Participant 10).

Second, beauty ideals increase young women’s negative emotions. Nearly half of the participants (*n* = 10, 41.67%) mentioned feeling anxious and two participants mentioned feeling envious after watching beauty ideals on social media. Several participants stated that they felt annoyed, unhappy, and sad after being exposed to beauty ideals on social media. For instance, Participant 5 stated:

“Sometimes the mood is suddenly bad. I wonder why my parents did not give me the perfect appearance. This puts me in a bad mood.”

Third, beauty ideals lead to an increase in inappropriate behavior among young women, mainly manifested as (1) spending time and energy to change their appearance, thereby affecting their study and life; (2) unhealthy weight loss and eating behaviors; (3) focusing on appearance; (4) increased frequency of appearance comparisons; (5) social avoidance; and (6) avoiding wearing clothes that make them look good. Participant 5 provided a more detailed description of this negative impact:

“They are determined to lose weight, but each time they manage to shed half of the weight, they cannot stick to it. Then, they cannot stick to eating a lot, feeling remorseful afterward and eating more. They wonder why they cannot be as self-disciplined and thin as the ideal women are. This impact is significant, I think. Consequently, I always think about how to make my appearance better. I also think about this problem during class and do not want to study. Every day, I think about this problem. As a result, I spend a lot of time and energy watching many videos that teach dress and makeup skills and rarely focus on studying.”

Fourth, beauty ideals negatively impact women’s body image. Twenty participants mentioned that they were dissatisfied with their bodies after seeing idealized images of women on social media. Three other participants reported feeling ashamed after seeing women with perfect features and great bodies on social media platforms. Further, one participant reported feeling anxious about her appearance after watching the videos. In addition, three participants mentioned that after watching such content, they deliberately controlled their diet and exhibited unhealthy eating behavior.

Finally, beauty ideals influence the values of young women, which was mainly reflected in the following aspects: (1) affecting and narrowing women’s understanding of beauty (*n* = 2, 8.33%); (2) making young women focus on their appearance rather than internal qualities (*n* = 4, 16.67%); (3) creating stereotypes of women, such as “women should be beautiful,” “women should learn makeup skills and dressing skills,” “women should not go out without makeup,” and “women’s appearance needs to meet the public aesthetic” (*n* = 3, 12.50%); (4) causing young women to internalize the concept of appearance having a high value (*n* = 2, 8.33%); and (5) weakening women’s internal value, enhancing their external value, and objectifying women (*n* = 1, 4.17%).

### 3.3 How do young Chinese women feel about body positive social media content?

All participants expressed their opinions on body positive social media content, including the existing problems and the path to improvement.

In terms of the existing problems, they first expressed concerns about and the professionalism of its creators (*n* = 5, 20.83%). They mentioned that although some body positive posts in social media are labeled as “#bodypositivity,” the core of the posts spreads beauty ideals. Participant 9 stated, “She told us to resist appearance anxiety, to refuse appearance anxiety, and that we cannot be the typical female beauty image; but at the same time, she taught us to put on makeup to create big eyes and double eyelids.”

Second, some participants also expressed concern about the heterogeneity of body positivity in content and the corresponding insufficience of persuasion (*n* = 11, 45.83%). For instance, bloggers’ posts before and after putting on makeup did not differ significantly. Observing such posts can make women unhappy with their bodies. Furthermore, bloggers with attractive features and figures are also involved in promoting body positivity; however, even when revealing their true appearance, they fall short of resonating with the experiences of ordinary women. No influential bloggers are dedicated to body positivity in China, and the movement is primarily championed by ordinary bloggers and young women. This contributes to a lack of widespread adoption of the body positivity movement on Chinese social media, as well as insufficient participation among women. In addition, participants also highlighted that the positive effects of body positive social media content were likely to be smaller for women with strong concerns about appearance, strong body dissatisfaction, or a fixed aesthetic (*n* = 3, 12.50%).

In terms of the path to improvement, some participants mentioned that although many women in China have been affected by poor body image and related eating disorders, this issue has not been seriously considered, and people appear unaware of the significance of the problem. To effectively leverage the body positivity movement for the improvement of Chinese women’s body image, additional efforts are needed. These include (1) finding professionals to lead the body positivity movement (*n* = 3, 12.50%); (2) conducting it both online and offline (*n* = 3, 12.50%); (3) focusing on girls and women between adolescence and early adulthood (*n* = 10, 41.67%); (4) increasing awareness of the concept, negative effects, and factors of negative body image while promoting positive body image (*n* = 4, 16.67%); and (5) promote the change of young parents’ views (*n* = 3, 12.50%).

### 3.4 What influences do young Chinese women think body positivity has?

Regarding exposure, except for one interviewee who did not participate in the body positivity movement, the remaining 23 participants had been exposed to body positive social media content and them expressed their opinions on the topic.

Twenty-three participants reported that body positive social media content has the potential to change women’s body image in the following ways. First, it helped young women realize that most people, with few exceptions, have an average appearance and that most young women suffer from appearance anxiety (common humanity) (*n* = 15, 62.50%). Participant 20 said,

“Although those bloggers are usually really good-looking in the videos, when we see them show their real appearance in the videos, we find that they also have round shoulders, small bellies, and meat on their legs, which motivate and make us think that even these beautiful girls have these shortcomings. I feel that there is no difference between myself and ordinary girls, and some of my shortcomings are acceptable. In addition, after seeing many bloggers share their experiences of body shame and appearance anxiety in videos, I realized that I am not the only one who suffers from appearance issues, and there are many others who suffer from this problem.”

Second, viewing body positive social media content increased women’s body satisfaction and decreased body shame (*n* = 14, 58.33%). For example, Participant 10 stated,

“There will certainly be an impact. We are ordinary people. We will not change too much, but after watching these contents, we become more confident, more satisfied with and accepting of ourselves. Everyone’s appearance is predetermined and difficult to change. These social media posts have made me realize that there are many kinds of beauty: white chrysanthemums look good, and pink roses look good, too. It is good for women and increases their confidence.”

Third, these contents encouraged women to accept themselves as they were (*n* = 12, 50.00%). Participant 23 said, “To accept yourself, you look like this, this is the way you are. To learn to accept it, you can change it in another way. To learn to accept the original self, you are like this.”

In addition, five participants mentioned that seeing body positive social media content increases women’s internal positivity. Seven participants stated that being exposed to body positive social media content helps women realize the diversity of beauty. One mentioned that watching such content helps women learn to take care of their bodies adaptively; another said that it helps women recognize the influence of negative information on the Internet.

The participants described the possible positive impact of body positivity on women; however, they noted that the spread of a large amount of such content on social media may also have negative effects on women. First, some participants were concerned that young women who oversubscribed to the idea and content of body positivity would not be motivated to improve themselves and would not focus on their appearance.

Additionally, they suggested that these women might have experienced negative emotions while revealing their perceived flaws. Participant 9 mentioned, “After being too accustomed to this concept, young women will not improve themselves, and it is difficult for them to make changes in appearance.”

## 4 Discussion

To the best of our knowledge, much of the current literature focuses on the impact of social media content on women’s body image. In fact, studies on young women’s perspectives on social media content, especially young Chinese women’s understanding of body positivity in social media, are scarce. This study aimed to explore the perspectives of young Chinese women regarding beauty ideals and body positivity in Chinese social media. A thematic analysis revealed four primary themes: (1) characteristics of mainstream beauty ideals in Chinese social media; (2) impact of beauty ideals on young women; (3) perspectives on the content of body positivity; (4) influences of body positive social media content on young women. Below is a separate discussion of each theme.

### 4.1 Characteristics of mainstream beauty ideals on Chinese social media

The findings suggest that young Chinese women perceived the idealized women’s image presented on Chinese social media as characterized by a thin, sexy, and curvy body, perfect features, flawless skin, fashionable attire, and delicate makeup. The pursuit of thinness aligns with a global trend among women in the pursuit of beauty ideals (Ando et al., 2021). Chinese women are partly influenced by the traditional stereotype of a soft and slender women being the most attractive, as well as the prevalence of thinspiration content in modern Chinese media (Jung, 2018). The pursuit of thinness leads to unhealthy weight loss and dieting behaviors in women, negatively impacting their physical and mental health (Jackson et al., 2021; Barnhart et al., 2022; McComb and Mills, 2022).

Furthermore, the beauty ideals in Chinese social media encourage women to pursue perfect features and fair, flawless skin, which can be seen in how young Chinese women select and adjust their photos and videos (Sun, 2021). However, numerous studies have shown that the pursuit of perfect features and skin, as well as the behavior of selfie-editing, leads to women’s facial dissatisfaction and cosmetic surgery consideration, as confirmed by the Chinese domestic beauty industry (Jackson et al., 2020; Sun, 2021; Wang et al., 2022; Wu et al., 2022). Additionally, the findings of current study revealed that many participants (*n* = 10, 41.67%) emphasized the concepts of “moderation and autonomy.” They underscored that young women can pursue thinness but not to the extreme of being overly slim. They also stressed that women have the freedom to obtain their desired physical features through methods such as cosmetic surgery but believe that they should not change their physical appearance merely to meet the relatively uniform and narrow standards of women’s beauty embedded in social culture and social media. Furthermore, women have a calibrated process of resisting physical ideals or pursuing certain ideals, and through this process, they keep themselves within the “healthy and appropriate” range (Ando et al., 2021). However, this calibration is rarely a flexible and healthy stance and rather one of constant effortful readjusting to perform “authenticity” (Cairns and Johnston, 2015). In addition, we can speculate that the moderation emphasized by the participants was not within the healthy range. Five of the 8 body mass indices of idealized female images provided by the participants were in the low weight range.

Moreover, the findings suggest that some content on Chinese social media encourages women to exercise to obtain a fit and sexy body. This content seems to be in line with the concept of fitspiration; however, our results show that young women in China tend to perceive this as an active and healthy lifestyle beneficial for women’s physical and mental health. Conversely, previous research has shown that fitspiration, similar to thinspiration, contains information about weight guilt, fat stigma, objectification of women, and dieting messages (Boepple and Thompson, 2016). All of these messages are associated with body dissatisfaction, increased negative emotions, and lower levels of physical self-esteem in women (Robinson et al., 2017; Alberga et al., 2018).

Furthermore, 50% of participants mentioned that the idealized women’s image was associated with personal characteristics, such as being energetic, elegant, and intellectual. This suggests that a diverse aesthetic appears to be budding, and the concept of looking at women from a “whole” perspective is increasing.

### 4.2 Impact of beauty ideals on young women

Consistent with the findings of previous studies, nearly all participants highlighted the challenge most women face in striving to attain mainstream ideal appearance standards promoted by the media through healthy means (Levine and Murnen, 2009). Moreover, they noted that the beauty ideals depicted on social media prompt women to invest excessive time and energy in chasing an idealized body image and effectively constrain them to a relatively limited spectrum of appearances, ultimately diminishing their focus on personal growth and achievement outside of the aesthetic sphere. This aligns with Roberts et al.’s (2018) discussion of the negative impact of self-objectification on women. According to Roberts et al. (2018), self-objectification conceals women’s potential, abilities, and autonomy, limiting their opportunities to expand themselves and their world by altering their aspirations and desires. This, in turn, narrows their life trajectories and experiences.

Surprisingly, some participants (*n* = 7, 29.17%) thought that beauty ideals positively encouraged women to learn makeup, underscoring that “moderate and autonomous involvement in the pursuit of ideal beauty is healthy.” However, the existing literature and the present study’s findings suggest that moderation may not truly signify health, but rather indicates that women have unconsciously internalized the concept of beauty ideals. This observation is consistent with the objectification theory and the tripartite influence model (Fredrickson and Roberts, 1997; Thompson and Heinberg, 1999).

### 4.3 Perspectives on the content and roles of body positivity

Our findings suggest that nearly a half of the participants expressed their concerns about the movement and women’s body image issues. First, the participants highlighted the possible issues with professionalism of body positive content on Chinese social media and the lack of specialized influencers, which is consistent with the content analysis results of Lang et al. (2023). Lang et al. (2023) found that over 40% of body positive posts on Chinese social media contained appearance-focused themes and that these body positive posts promoted ideas consistent with thinspiration and fitspiration, urging women to pursue prevailing beauty standards embedded in social culture (Cohen et al., 2019; Lazuka et al., 2020; Lang et al., 2023).

Moreover, the participants expressed that some women with perfect facial features and figures participate in the body positivity movement, a factor that fails to comfort other women but instead fosters dissatisfaction and anxiety about their own bodies. This may be attributed to the phenomenon where young women, upon seeing such posts, engage in upward appearance comparisons, resulting in negative emotions such as low self-esteem, frustration, shame, and anxiety (Alberga et al., 2018; Yang et al., 2020). Long-term active social media users, known as influencers, are generally women with higher levels of physical attractiveness than the average; thus, their body positive posts are more prominently visible. Therefore, even if only a few bloggers conforming to mainstream beauty standards participate in the body positivity movement, participants feel the influence of such bloggers in spreading the movement’s message (Harriger et al., 2023). Furthermore, the participants believed that women with high levels of trait body satisfaction or a stable self image aesthetically might be less likely to be affected by positive social media content, which may need to be explored in future studies. According to the current literature, the examination of body positive content mainly focuses on young women, but not enough on the characteristics of the individuals themselves (Rodgers et al., 2022). A recent review suggested elucidating the interaction between personal characteristics and body positive social media content to understand which types of content are more useful and to whom (Rodgers et al., 2022).

In addition, 11 participants offered ways to improve the effect of the movement of body positivity, with interventions for female middle and high school students being the most frequently mentioned (*n* = 10). Nine participants reported that they suffered from a lot of body image disturbers during adolescence and worked hard to overcome it. They believe that if they can embrace the concept of body positivity during adolescence, they may be able to get through that period more easily. This is consistent with previous literature, which has found that the transition from adolescence to early adulthood is the most prominent stage of development of body image disorder (Bucchianeri et al., 2013). Additionally, another participant mentioned that she was exposed to and internalized the idea of body positivity in high school, so she was not obviously influenced by beauty ideals in social media. This, in turn, is consistent with Tylka and Wood-Barcalow's (2015) discussion of positive body image. Another study with a sample of 567 Chinese female college students also showed that body appreciation significantly moderated the relationship between social media body image comparison and body shame and restrictive eating behaviors (Yao et al., 2021). Besides, some participants mentioned ways of exposing young parents to the concept of body positivity and having professionals lead body positivity movement, which could be considered in future interventions.

### 4.4 Influence of body positive social media content on young women

The results suggest that young Chinese women believe that positive social media reveals the falsity of “perfect” women on social media through posts showing images before and after makeup and retouching. They also realize that most women who meet mainstream beauty standards on social media have shortcomings in their appearance and that everyone is “ordinary.” Therefore, watching such content may reduce women’s general dissatisfaction with and anxiety about their bodies. By watching body positive social media content, young Chinese women reported their awareness of the harm of pursuing beauty ideals, the importance of taking adaptive care of their bodies, and maintaining internal positivity. Moreover, the participants stressed that body positive social media content made them see the diversity of women’s beauty and want to pursue their own unique beauty. These results indicate that the benefits of body positivity perceived by young Chinese women are consistent with the main thrust of the body positivity movement, which argues that the core concepts conveyed and expressed by body positive social media content are recognized and accepted by its users. In addition, the present results are consistent with a previous content analysis that found that body positive posts on Chinese social media contain both the core concept of positive body image and information consistent with the concept of self-compassion (Lang et al., 2023). Thus, positive body content on social media could help women improve their body image and promote a positive one (Rodgers et al., 2022).

However, the participants also expressed concerns about whether frequent exposure to body positive social media content would diminish their motivation to manage their self care. No adverse results have been reported in the literature, and previous studies have found that viewing such content does not cause women to choose more caloric foods or overeat (Simon and Hurst, 2021; Rodgers et al., 2022). Therefore, we speculate that body positive social media content is unlikely to be detrimental to women’s self-management; however, further research is warranted to confirm this hypothesis. Further, participants voiced their concerns on creators of body positive social media content, particularly those who are plus-sized or diverge from mainstream beauty standards, who may receive malicious comments or accusations when sharing such content, which could potentially lead to dissatisfaction with their own appearance. Nonetheless, this concern necessitates further investigation.

### 4.5 Limitations and future directions

Our study had several limitations. First, all participants were female college students whose views may not necessarily represent those of all young women. In several interviews, participants mentioned that middle and high school were the periods when they experienced the highest levels of body dissatisfaction. Therefore, future research could expand the sample diversity to include adolescent girls or other women’s groups to gain a broader and deeper understanding of women’s perspectives on social media content. Second, no demographic variables other than age were collected from the participants, limiting the scope of our findings’ generalization. Body positive social media content is heterogeneous, and women may respond differently to different types of body positive content (Rodgers et al., 2023). A previous study suggested that body positive posts that highlight the unrealistic nature of social media and are inclusive in their broad portrayals of beauty were the most helpful; however, these effects may vary according to individuals’ own body image characteristics and their proximity to content creators from the body positivity movement (Rodgers et al., 2023). Hence, the demographic variables of individuals should be expanded in future studies to identify the most effective types of body positivity for different individuals (Rodgers et al., 2022, 2023). Finally, we found that some participants viewed social media content promoting fitspiration as endorsing an active and healthy lifestyle beneficial to women’s physical and mental health. Given previous research highlighting concerns about the negative effects of fitspiration on women’s body image, women’s perceptions of this type of content should be clarified in future research (Alberga et al., 2018; Fioravanti et al., 2021).

### 4.6 Implications

This study provides several implications. First, considering the impact of social media’s beauty ideals on women’s well-being, educators and parents should guide girls to consciously and critically engage with social media and strategically avoid harmful content by unfollowing or blocking accounts, users, and sites (Hockin-Boyers et al., 2020). Second, considering the benefits of body positive social media content, the findings suggest that body image researchers and health professionals should incorporate body positive social media content into preventive or therapeutic practices. Finally, considering young Chinese women’s concerns about the professionalism of body positive content creators on Chinese social media, as well as the results indicating that often such content focuses on appearance, it is essential to ensure that body positive social media content aligns with the main thrust of the body positivity movement (Lang et al., 2023). If researchers and health professionals, particularly those who are most familiar with women’s body image issues, participate in the body positivity movement in this digital era, their expertise may lead the body positivity movement in a more professional and beneficial direction. Moreover, the population benefiting from their specialized knowledge and expertise would significantly expand (Sharp et al., 2023).

## 5 Conclusion

Previous research has established an association between Asian media, poor body image, plastic surgery, and dieting behaviors among young Chinese women (Jackson et al., 2020; Wang et al., 2023). The present study used qualitative research methods to examine young Chinese women’s perspectives on beauty ideals and body positive content on social media and its effects on women’s body image. Our findings indicate that young Chinese women perceive the pressure of social media not only from traditional Chinese culture but also due to the globalization of Western culture. Moreover, young Chinese women expressed that the beauty ideals promoted by social media were less extreme than traditional ones, reminding people to pursue beauty ideals in moderation. However, the findings suggested that this moderation did not necessarily mean remaining within the moderate range suggested by health professionals. In addition, most participants understood that beauty ideals on social media negatively influenced them and that they sought to change and adjust.

Most young Chinese women have been exposed to body positive social media content. Given its role in increasing women’s body satisfaction and bridging the gap between their own and others’ appearances online, young Chinese women believe that such content can have the potential to improve women’s body image. However, considering the lack of creators’ professionalism in some body positive content on social media, future improvement of body positivity movements requires consideration. Finally, considering the heterogeneity of body positive social media content, future research should identify the types of content that are most effective for people with specific characteristics.

## Data availability statement

The raw data supporting the conclusions of this article will be made available by the authors, without undue reservation.

## Ethics statement

The studies involving humans were approved by Fujian Normal University Ethics Committee. The studies were conducted in accordance with the local legislation and institutional requirements. The participants provided their written informed consent to participate in this study.

## Author contributions

ML: Writing – review & editing, Writing – original draft, Supervision, Methodology, Investigation, Formal analysis, Data curation, Conceptualization. YY: Writing – review & editing, Supervision, Methodology, Formal analysis.

## References

[ref1] Alberga A. S. Withnell S. J. von Ranson K. M. (2018). Fitspiration and thinspiration: a comparison across three social networking sites. J. Eat. Disord. 6:39. doi: 10.1186/s40337-018-0227-x, PMID: 30534376 PMC6260773

[ref2] Ando K. Giorgianni F. E. Danthinne E. S. Rodgers R. F. (2021). Beauty ideals, social media, and body positivity: a qualitative investigation of influences on body image among young women in Japan. Body Image 38, 358–369. doi: 10.1016/j.bodyim.2021.05.001 34120098

[ref3] Barnhart W. R. Cui T. Cui S. Han X. Lu C. He J. (2022). Examining appearance pressures, thinness and muscularity internalizations, and social comparisons as correlates of drive for muscularity and thinness-oriented disordered eating in Chinese heterosexual men and women: testing an integrated model. Body Image 43, 429–439. doi: 10.1016/j.bodyim.2022.10.005, PMID: 36345081

[ref4] Boepple L. Thompson J. K. (2016). A content analytic comparison of fitspiration and thinspiration websites. Int. J. Eat. Disord. 49, 98–101. doi: 10.1002/eat.22403, PMID: 25778714

[ref5] Braun V. Clarke V. (2021). Thematic analysis: a practical guide: SAGE Publications Available at: https://books.google.com.au/books?id=mToqEAAAQBAJ.

[ref6] Bucchianeri M. M. Arikian A. J. Hannan P. J. Eisenberg M. E. Neumark-Sztainer D. (2013). Body dissatisfaction from adolescence to young adulthood: findings from a 10-year longitudinal study. Body Image 10, 1–7. doi: 10.1016/j.bodyim.2012.09.001, PMID: 23084464 PMC3814026

[ref7] Cairns K. Johnston J. (2015). Choosing health: embodied neoliberalism, postfeminism, and the “do-diet”. Theory Soc. 44, 153–175. doi: 10.1007/s11186-015-9242-y

[ref8] Chen H. S. (1995). On the description of female form in classical poetry. Jianghan Luntan 10, 79–85,

[ref9] Clarke V. Braun V. (2013). Successful Qualitative Research: A Practical Guide for Beginners London: Sage, 382.

[ref10] Cohen R. Fardouly J. Newton-John T. Slater A. (2019). #BoPo on Instagram: an experimental investigation of the effects of viewing body positive content on young women’s mood and body image. New Media Soc. 21, 1546–1564. doi: 10.1177/1461444819826530

[ref11] Cohen R. Irwin L. Newton-John T. Slater A. (2019). #Bodypositivity: a content analysis of body positive accounts on Instagram. Body Image 29, 47–57. doi: 10.1016/j.bodyim.2019.02.007, PMID: 30831334

[ref12] Cwynar-Horta J. (2016). The commodification of the body positive movement on Instagram. Stream 8, 36–56. doi: 10.21810/strm.v8i2.203

[ref13] De Valle M. K. Gallego-García M. Williamson P. Wade T. D. (2021). Social media, body image, and the question of causation: Meta-analyses of experimental and longitudinal evidence. Body Image 39, 276–292. doi: 10.1016/j.bodyim.2021.10.001, PMID: 34695681

[ref14] Fioravanti G. Svicher A. Ceragioli G. Bruni V. Casale S. (2021). Examining the impact of daily exposure to body-positive and fitspiration Instagram content on young women’s mood and body image: an intensive longitudinal study. New Media Soc. 25, 3266–3288. doi: 10.1177/14614448211038904

[ref15] Forbes M. (2022). Thematic analysis: a practical guide. Evaluat. J. Aust. 22, 132–135. doi: 10.1177/1035719X211058251

[ref16] Fredrickson B. L. Roberts T. A. (1997). Objectification theory: toward understanding women's lived experiences and mental health risks. Psychol. Women Qtrly. 21, 173–206. doi: 10.1111/j.1471-6402.1997.tb00108.x

[ref17] Gattario K. H. Frisén A. (2019). From negative to positive body image: Men's and women's journeys from early adolescence to emerging adulthood. Body Image 28, 53–65. doi: 10.1016/j.bodyim.2018.12.002 30583277

[ref18] Guo L. Gu L. Peng Y. Gao Y. Mei L. Kang Q.. (2022). Online media exposure and weight and fitness management app use correlate with disordered eating symptoms: evidence from the mainland of China. J. Eat. Disord. 10:58. doi: 10.1186/s40337-022-00577-y, PMID: 35468844 PMC9036716

[ref19] Harriger J. A. Wick M. R. Sherline C. M. Kunz A. L. (2023). The body positivity movement is not all that positive on TikTok: a content analysis of body positive TikTok videos. Body Image 46, 256–264. doi: 10.1016/j.bodyim.2023.06.003 37379612

[ref20] Hockin-Boyers H. Pope S. Jamie K. (2020). Digital pruning: agency and social media use as a personal political project among female weightlifters in recovery from eating disorders. New Media Soc. 23, 2345–2366. doi: 10.1177/1461444820926503

[ref21] Holmqvist K. Frisén A. (2012). "I bet they aren't that perfect in reality:" appearance ideals viewed from the perspective of adolescents with a positive body image. Body Image 9, 388–395. doi: 10.1016/j.bodyim.2012.03.007, PMID: 22542634

[ref22] Hong'e M. (2017). China now world's fastest growing plastic surgery market. Retrieved from International Society for Aesthetic Plastic Surgery (2013). ISAPS International Survey on Aesthetic/Cosmetic Procedures Performed in 2010. Available at: https://www.isaps.org/wp-content/uploads/2017/10/ISAPS-Results-Procedures-2010-1.pdf

[ref23] Jackson T. Cai L. Chen H. (2020). *Asian versus Western* appearance media influences and changes in body image concerns of young Chinese women: a 12-month prospective study. Body Image 33, 214–221. doi: 10.1016/j.bodyim.2020.03.008, PMID: 32361154

[ref24] Jackson T. Ye X. Hall B. J. Chen H. (2021). "Have you taken the A4 challenge?" correlates and impact of a thin ideal expression from Chinese social media. Front. Psychol. 12:669014. doi: 10.3389/fpsyg.2021.669014, PMID: 34163409 PMC8215108

[ref25] Jung J. (2018). Young Women's perceptions of traditional and contemporary female beauty ideals in China. Fam. Consum. Sci. Res. J. 47, 56–72. doi: 10.1111/fcsr.12273

[ref26] Lang M. Chen P. Li X. Ye Y. (2023). “Refusing appearance anxiety”: a content analysis of body positive posts on Chinese social media. Body Image 45, 414–419. doi: 10.1016/j.bodyim.2023.04.002, PMID: 37146442

[ref27] Lazuka R. F. Wick M. R. Keel P. K. Harriger J. A. (2020). Are we there yet? Progress in depicting diverse images of beauty in Instagram’s body positivity movement. Body Image 34, 85–93. doi: 10.1016/j.bodyim.2020.05.001, PMID: 32534269

[ref28] Levine M. P. Murnen S. K. (2009). "Everybody knows that mass media are/are not [pick one] a cause of eating disorders": a critical review of evidence for a causal link between media, negative body image, and disordered eating in females. J. Soc. Clin. Psychol. 28, 9–42. doi: 10.1521/jscp.2009.28.1.9

[ref30] McComb S. E. Mills J. S. (2022). Eating and body image characteristics of those who aspire to the slim-thick, thin, or fit ideal and their impact on state body image. Body Image 42, 375–384. doi: 10.1016/j.bodyim.2022.07.017, PMID: 35930873

[ref32] New Oxygen. 2021 white paper on medical beauty. (2021). Available at: https://www.soyoung.com/p31394916/

[ref33] Niu G. F. Sun L. Liu Q. Chai H. Sun X. J. Zhou Z. (2020). Selfie-posting and young adult Women’s restrained eating: the role of commentary on appearance and self-objectification. Sex Roles 82, 232–240. doi: 10.1007/s11199-019-01045-9

[ref37] Quan M. (2021). A study on the impact of Douyin’s “ideal beauty” presentation on female college students’ body image, (Unpublished master’s thesis). Xi ‘an, China: Northwest University.

[ref38] Roberts T. A. Calogero R. M. Gervais S. J. (2018). “Objectification theory: continuing contributions to feminist psychology” in APA handbook of the psychology of women: History, theory, and battlegrounds. eds. Travis C. B. White J. W. Rutherford A. Williams W. S. Cook S. L. Wyche K. F. (Washington: American Psychological Association), 249–271.

[ref39] Robinson L. Prichard I. Nikolaidis A. Drummond C. Drummond M. Tiggemann M. (2017). Idealised media images: the effect of fitspiration imagery on body satisfaction and exercise behaviour. Body Image 22, 65–71. doi: 10.1016/j.bodyim.2017.06.001, PMID: 28654826

[ref40] Rodgers R. F. Laveway K. Zalvino J. Cardone W. Wang L. (2023). #BodyPositive: a qualitative exploration of young people’s responses to body positive social media content. Body Image 47:101613. doi: 10.1016/j.bodyim.2023.08.005, PMID: 37659247

[ref41] Rodgers R. F. Paxton S. J. Wertheim E. H. (2021). #take idealized bodies out of the picture: a scoping review of social media content aiming to protect and promote positive body image. Body Image 38, 10–36. doi: 10.1016/j.bodyim.2021.03.009, PMID: 33798800

[ref42] Rodgers R. F. Wertheim E. H. Paxton S. J. Tylka T. L. Harriger J. A. (2022). #Bopo: enhancing body image through body positive social media- evidence to date and research directions. Body Image 41, 367–374. doi: 10.1016/j.bodyim.2022.03.008, PMID: 35525155

[ref43] Sharp G. Bilal M. Fernando A. N. de Boer K. (2023). Examining health professional perspectives on social media body image movements: a qualitative exploration. Body Image 46, 230–237. doi: 10.1016/j.bodyim.2023.06.004, PMID: 37364499

[ref44] Simon K. Hurst M. (2021). Body positivity, but not for everyone: the role of model size in exposure effects on women's mood, body satisfaction, and food choice. Body Image 39, 125–130. doi: 10.1016/j.bodyim.2021.07.001 34333414

[ref45] South China Morning Post. Report: More than 70 percent of women are dissatisfied with their appearance. (2020).

[ref46] Stevens A. Griffiths S. (2020). Body positivity (#BoPo) in everyday life: an ecological momentary assessment study showing potential benefits to individuals' body image and emotional wellbeing. Body Image 35, 181–191. doi: 10.1016/j.bodyim.2020.09.003 33068893

[ref47] Sun Q. (2021). Selfie editing and consideration of cosmetic surgery among young Chinese women: the role of self-objectification and facial dissatisfaction. Sex Roles 84, 670–679. doi: 10.1007/s11199-020-01191-5

[ref49] Thompson J. K. Heinberg L. J. (1999). The media's influence on body image disturbance and eating disorders: We've reviled them, now can we rehabilitate them? Oxford: Blackwell Publishing.

[ref50] Tiggemann M. Anderberg I. (2019). Social media is not real: the effect of ‘Instagram vs reality’ images on women’s social comparison and body image. New Media Soc. 22, 2183–2199. doi: 10.1177/1461444819888720

[ref54] Tylka T. L. Wood-Barcalow N. L. (2015). What is and what is not positive body image? Conceptual foundations and construct definition. Body Image 14, 118–129. doi: 10.1016/j.bodyim.2015.04.001, PMID: 25921657

[ref55] Wang Y. Chu X. Nie J. Gu X. Lei L. (2022). Selfie-editing, facial dissatisfaction, and cosmetic surgery consideration among Chinese adolescents: a longitudinal study. Curr. Psychol. 41, 9027–9037. doi: 10.1007/s12144-020-01280-4

[ref56] Wang Y. Qiao X. Yang J. Geng J. Fu L. (2023). "I wanna look like the person in that picture": linking selfies on social media to cosmetic surgery consideration based on the tripartite influence model. Scand. J. Psychol. 64, 252–261. doi: 10.1111/sjop.12882 36321668

[ref57] Wu J. Lin Z. Liu Z. He H. Bai L. Lyu J. (2022). Secular trends in the incidence of eating disorders in China from 1990 to 2017: a joinpoint and age-period-cohort analysis. Psychol. Med. 52, 946–956. doi: 10.1017/s0033291720002706 32744194

[ref58] Wu Y. Mulkens S. Alleva J. M. (2022). Body image and acceptance of cosmetic surgery in China and the Netherlands: a qualitative study on cultural differences and similarities. Body Image 40, 30–49. doi: 10.1016/j.bodyim.2021.10.007 34801810

[ref59] Yang J. Fardouly J. Wang Y. Shi W. (2020). Selfie-viewing and facial dissatisfaction among emerging adults: a moderated mediation model of appearance comparisons and self-objectification. Int. J. Environ. Res. Public Health 17:672. doi: 10.3390/ijerph17020672, PMID: 31968671 PMC7013747

[ref60] Yao L. Niu G. Sun X. (2021). Body image comparisons on social networking sites and Chinese female college students’ restrained eating: the roles of body shame, body appreciation, and body mass index. Sex Roles 84, 465–476. doi: 10.1007/s11199-020-01179-1

[ref61] Zhang L. Qian H. Fu H. (2018). To be thin but not healthy - the body-image dilemma may affect health among female university students in China. PLoS One 13:e0205282. doi: 10.1371/journal.pone.0205282, PMID: 30304026 PMC6179281

