# Supplementary material for: Beauty ideals and body positivity: a qualitative investigation of young women’s perspectives on social media content in China
Source: Front Psychol. 2024 May 20;15:1389935. doi: 10.3389/fpsyg.2024.1389935 (PMC11144859; doi:10.3389/fpsyg.2024.1389935)
Supplement: Supplementary file 1 [file Data_Sheet_1.DOCX]

Supplementary Material

Guiding questions

1. In your opinion, what is the idealized appearance of women currently portrayed in social media?
2. What influence do you think these idealized women images have on women?
3. Have you ever seen a post on social media with the hashtag #bodypositivity? How do you feel when you browse these posts?
4. What are the effects of #bodypositivity posts on women? Do you think body positivity in social media has the potential to improve women's body image?
